# Validación clínica de la prueba RT-LAMP para el diagnóstico rápido del SARS-CoV-2

**DOI:** 10.7705/biomedica.6523

**Published:** 2022-10-31

**Authors:** Leidy Hurtado, Diana Díaz, Katherine Escorcia, Laura Flórez, Yesit Bello, Yirys Díaz, Elkin Navarro, Leonardo C. Pacheco, Nataly Galán, Ronald Maestre, Antonio Acosta, Lisandro A. Pacheco

**Affiliations:** 1 División Ciencias de la Salud, Universidad del Norte, Barranquilla, Colombia Universidad del Norte Universidad del Norte Barranquilla Colombia; 2 Programa de Microbióloga, Universidad Simón Bolívar, Barranquilla, Colombia Universidad Simón Bolívar Universidad Simón Bolívar Barranquilla Colombia; 3 Unidad de Genética y Biología Molecular, Facultad de Ciencias Básicas y Biomédicas, Universidad Simón Bolívar, Barranquilla, Colombia Universidad Simón Bolívar Universidad Simón Bolívar Barranquilla Colombia; 4 Centro de Investigaciones en Ciencias de la Vida, Universidad Simón Bolívar, Barranquilla, Colombia Universidad Simón Bolívar Universidad Simón Bolívar Barranquilla Colombia; 5 Facultad de Ciencias de la Salud, Universidad Simón Bolívar, Barranquilla, Colombia Universidad Simón Bolívar Universidad Simón Bolívar Barranquilla Colombia

**Keywords:** COVID-19/diagnóstico, técnicas de diagnóstico molecular, sensibilidad y especificidad, pruebas en el punto de atención, COVID-19/diagnosis, molecular diagnostic techniques, sensitivity and specificity, point-of-care testing

## Abstract

**Introducción.:**

Desde el primer reporte en la provincia de Wuhan (China) en el año 2019, el SARS-CoV-2 se ha diseminado por todo el mundo, provocando un enorme impacto en la salud pública. Para su diagnóstico, la Organización Mundial de la Salud ha incentivado el desarrollo de pruebas rápidas, de simple ejecución, sensibles y específicas, que complementan la RT-qPCR como prueba de referencia. La prueba RT-LAMP ha mostrado ser una excelente alternativa para la detección del SARS-CoV-2 en diferentes biofluidos.

**Objetivo.:**

Validar la técnica RT-LAMP colorimétrica en muestras de hisopado nasofaríngeo previamente confirmadas por RT-qPCR, usando el protocolo Charité, Berlín, Alemania.

**Materiales y métodos.:**

Un total de 153 muestras de hisopado nasofaríngeo de individuos con sospecha de COVID-19 se sometieron a RT-qPCR y RT-LAMP, usando un estuche comercial colorimétrico (NEB, Germany). La RT-LAMP se practicó con las muestras de ARN extraídas del hisopado nasofaríngeo y con muestras crudas sin previa extracción de ARN. El resultado fue evaluado por un simple cambio de color en la reacción.

**Resultados.:**

La sensibilidad y especificidad de la técnica RT-LAMP para detectar el gen *N* del SARS-CoV-2 mediante un set de cebadores previamente reportados (set de Broughton), arrojó valores de 0,97 (0,85-1,00) y 0,81 (0,65-0,92), respectivamente, con un intervalo de confianza del 95%. Otro set de cebadores dirigidos contra otra región del mismo gen (set de Lalli) arrojó valores de sensibilidad y especificidad de 0,96 (0,78-1,00) y 0,77 (0,55-0,92), respectivamente. Sin previa extracción de ARN, se encontró que la sensibilidad fue del 0,95 (0,74-1,00) y la especificidad del 0,88 (0,64-0,99).

**Conclusiones.:**

Estos resultados evidencian que la técnica RT-LAMP podría considerarse una prueba diagnóstica rápida, de fácil ejecución, libre de equipos sofisticados, sensible y específica, para el diagnóstico del SARS-CoV-2 en muestras de hisopados nasofaríngeos.

## Abstract (en)

**Introduction:**

Since the first report in Wuhan (China) in 2019, the SARS-CoV-2 virus has spread throughout the world, with a significant impact in public health. To contain its transmission, the WHO has encouraged the development of rapid, simple, sensitive and specific tests that complement qRT-PCR, as the gold standard. RT-LAMP has shown to be a good alternative to detect SARS-CoV-2 in different fluid samples.

**Objective:**

To validate the colorimetric RT-LAMP technique using two sets of primers targeting *N* gene of SARS-CoV-2 in 117 nasopharyngeal swab samples previously confirmed by RT-qPCR, using the Charité/Berlin protocol.

**Material and methods:**

A total of 153 nasopharyngeal swab samples from individuals with suspected COVID-19 were subjected to qRT-PCR and RT-LAMP using a commercial colorimetric kit (NEB, Germany). RT-LAMP was performed using both extracted RNA samples and raw samples without prior RNA extraction, and the result was assessed by a simple color change in the reaction.

**Results:**

Sensitivity and specificity for the previously reported RT-LAMP primers (Broughton set) targeting *N* gene of SARS-CoV-2 were 0.97 (0.85-1.00) and 0.81 (0.65-0.92) respectively, with CI95%. The Lalli primers targeting another region of the *N* gene used showed a sensitivity value of 0.96 (0.78-1.00) and a specificity of 0.77 (0.55-0.92). Without RNA extraction we found a sensitivity value of 0.95 (0.74, 1.00) and a specificity of 0.88 (0.64, 0.99). A sensitivity value of 0.95 (0.74-1.00) and a specificity 0.88 (0.64-0.99) were found without prior RNA extraction.

**Conclusion:**

Taking together, the results showed that RT-LAMP technique could be considered as a rapid diagnostic test, easy to perform, free of sophisticated equipment, sensitive and specific to diagnose SARS-CoV-2 in nasopharyngeal swabs with and without prior RNA extraction, allowing its implementation in places with scarce resources.

La aparición de un nuevo síndrome respiratorio agudo y grave (COVID-19), ocasionado por el virus SARS-CoV-2, se ha desarrollado como un evento pandémico que produjo graves problemas de salud pública, con un amplio impacto en la economía global. Desde el inició de la pandemia en diciembre del 2019 hasta agosto del 2022, la COVID-19 ha sido responsable de más de 6,4 millones de muertes y sobrepasa los 600 millones de personas infectadas a nivel mundial desde el primer reporte en la provincia de Wuhan, China ^1^.

Es de resaltar que el SARS-CoV-2 es un virus perteneciente a la familia Coronaviridae, género *Betacoronavirus* y subgénero *Sarbecoronavirus* o *Sarbecovirus*^2^.

El diagnóstico molecular del SARS-CoV-2 se basa en la detección de proteínas virales en pruebas serológicas o de antígenos, o a partir del ARN viral en muestras clínicas de individuos con sospecha de COVID-19. Las pruebas de antígenos se consideran como las menos sensibles y con menor especificidad para diagnosticar la enfermedad ^3^.

En la actualidad, la reacción en cadena de la polimerasa en tiempo real con transcriptasa inversa (RT-qPCR) se considera la técnica molecular más sensible y específica para diagnosticar el SARS-CoV-2 ^4^. Sin embargo, la RT-qPCR es una técnica compleja y laboriosa que requiere de equipos eficientes y costosos, y de personal entrenado e infraestructura específica para su ejecución. En concordancia con la aparición de nuevas variantes del virus con un potencial de diseminación mucho mayor a la cepa original del SARS-CoV-2, la Organización Mundial de la Salud (OMS) está incentivando el desarrollo de nuevas herramientas de diagnóstico molecular que soslayen las dificultades antes mencionadas de la RT-qPCR, y que mantengan su buen grado de sensibilidad y especificidad.

Algunas metodologías de diagnóstico molecular han surgido en los últimos tiempos, como RT-LAMP, DETECTR y SHERLOCK (basado en el sistema CRISPR/Cas) ^5^, y amplificación por polimerasas y recombinasas (RPA), entre otras ^6^^-^^10^, todas con sensibilidad y especificidad variables. De todas las metodologías anteriormente mencionadas, la técnica RT-LAMP ha demostrado un enorme potencial en el diagnóstico de SARS-CoV-2 y podría ser una metodología útil para complementar el diagnóstico de COVID-19 en centros de salud con bajos recursos.

La técnica molecular LAMP (*Loop Mediated Isothermal Amplification*) es una prueba de principio isotérmico; es decir, se ejecuta a una única temperatura. Por lo tanto, las pruebas diagnósticas basadas en ella pueden practicarse en cualquier lugar, inclusive en aquellos con recursos limitados, debido a que solo se requiere un equipo que pueda ser ajustado a una única temperatura. Los abordajes colorimétricos de la LAMP incluyen la detección de la turbidez de la reacción, provocada por la acumulación de pirofosfato de magnesio ^10^, y también, cambios de color que ocurren cuando colorantes sensibles a cambios de pH o incluso, colorantes interpuestos de ADN, se incorporan en la reacción ^11^^-^^14^ (figura 1 y 2).

**Figura 1:**
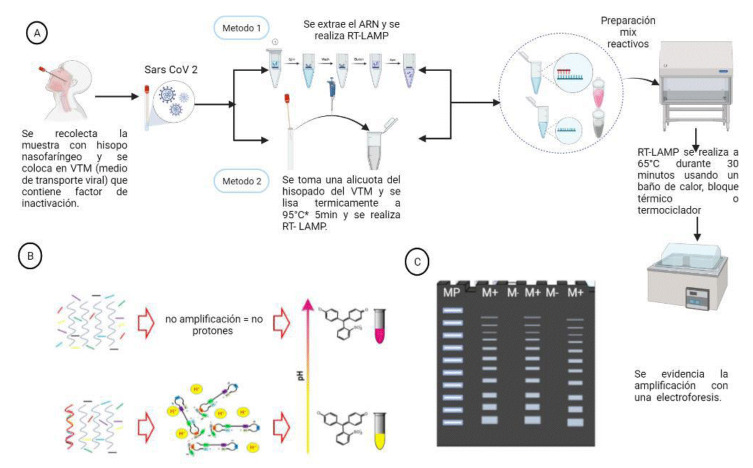
Flujograma conceptual de la prueba LAMP para detectar SARS-CoV-2 en muestras de hisopado nasal. A) La reacción de RT-LAMP a partir de muestras de hisopado nasofaríngeo de individuos con sospecha de Covid-19 se ejecuta a partir de: i) ARN extraído o ii) directamente de las muestras sin extracción de ARN, con un previo paso de inactivación a 95 °C por 5 minutos. B) La RT-LAMP hace uso de 6 cebadores (marcados aquí en colores) que, en presencia del ARN diana (espiral de color rojo), inician la amplificación isotérmica, generando protones que inducen un cambio de pH en la reacción, con la subsecuente virada de color de rosa a amarillo. C) La reacción de amplificación en RT-LAMP genera productos de diferentes masas moleculares en forma de concatámeros que pueden observarse fácilmente en un gel de agarosa.

Figura creada usando el *software* Biorender.

**Figura 2:**
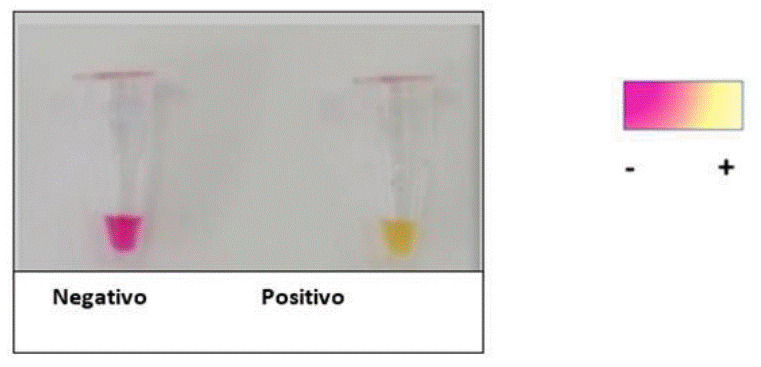
Resultado colorimétrico de RT-LAMP. Reacción color rosa: muestra negativa para SARS-CoV-2. Reacción color amarillo: resultado positivo para el gen N del SARS-CoV-2

En la técnica LAMP se emplea una ADN polimerasa termoestable, conocida como Bst, aislada de la bacteria *Bacillus stearothermophilus*, cuya actividad enzimática se da a temperaturas entre 60 y 65 °C, y un conjunto de 4 a 6 cebadores que permite identificar 6 a 8 secuencias distintas en el gen diana de interés; una DNA polimerasa con actividad de desplazamiento de hebra inicia la síntesis y dos de los cebadores forman estructuras en bucle para facilitar las subsecuentes rondas de amplificación ^15^ (figura 1B).

Esta ADN polimerasa tiene actividad helicasa y actividad exonucleasa 3-5' ^16^, además de ser mucho más robusta que la Taq ADN polimerasa (*Thermus aquaticus*), empleada en la PCR convencional, debido a que la Bst puede actuar normalmente en presencia de inhibidores presentes en muestras biológicas o medios de transporte viral, que habitualmente interfieren con el desempeño de la técnica. Cuando la diana que se quiere amplificar es de ARN, se incorpora una transcriptasa inversa a la reacción, por lo cual se denomina RT-LAMP.

En el presente trabajo, se evalúa el desempeño de la prueba isotérmica RT-LAMP a partir de ARN extraído de muestras nasofaríngeas de pacientes con sospecha de COVID-19, remitidas a la Unidad de Genética y Biología Molecular de la Universidad Simón Bolívar de Barranquilla. De igual forma, se reportan los resultados preliminares de la detección del ARN viral por RT- LAMP directamente en hisopados nasofaríngeos sin previa extracción de ARN.

## Materiales y métodos

### Muestras

Se recolectaron 153 muestras de hisopados nasofaríngeos de pacientes con sospecha de COVID-19, en la Unidad de Genética y Biología Molecular de la Universidad Simón Bolívar de Barranquilla, durante el período comprendido entre junio de 2021 y enero de 2022. Las muestras se inactivaron rápidamente en medio de transporte viral y se almacenaron a -80 °C hasta su uso.

### Extracción de ARN

De ellas, 117 muestras se sometieron a extracción de ARN a partir de 200 μΙ de hisopado nasofaríngeo contenido en medio de transporte viral, con el kit de extracción Quick-RNA Viral Kit™ (ZYMO Research), que emplea perlas magnéticas de sílice para la purificación del ARN total, siguiendo las recomendaciones del fabricante. En seguida, se adicionaron 400 μΙ de solución tampón de ARN viral a cada 200 μΙ de muestra (2:1) y se mezcló suavemente. Posteriormente, se realizaron lavados sucesivos para, finalmente, eluir el ARN en 15 μl de agua libre de ADNasa/RNasa.

### RT-qPCR

Para llevar a cabo la RT-PCR, se utilizó el kit SuperScript III One-Step RT-PCR System with Platinum Taq DNA Polymerase™ (Invitrogen, catálogo: 1257401). Se emplearon 8 μl de solución tampón de reacción 2X que contenía 0,4 mM de cada dNTP, 3,2 mM de MgSO_4_, 0,3 μl de transcriptasa inversa (SuperScript III RT/Platinum Taq Mix), 200 nM de cada cebador dirigido contra el gen *E* del SARS-CoV-2 y 100 nM de sonda TaqMan conjugada al fluoróforo CAL Red 610. Además, se introdujo un control endógeno a la reacción (gen RNase P humano), 100 nM de cada cebador y 100 nM de sonda TaqMan-Hex, para su amplificación. Los cebadores empleados para amplificar un fragmento del gen E que codifica para la envoltura del virus (específico del subgénero *Sarbecovirus*) fueron proporcionados por Biosearch Technologies™, con base en el protocolo descrito por Corman, *et al*. ^17^. Las secuencias se muestran en el cuadro 1.

**Cuadro 1:** Cebadores utilizados en este trabajo para la detección colorimétrica del gen N de SARS-CoV-2

Cebadores para amplificación	[ ] Inicial (10X)		[ ] en la LAMP (1X)	Set de iniciadores	
				Secuencia 5'-3' Gen N SARS-CoV-2	
				**Broughton, *et al.*** ^1^	**Lalli, *et al*.**^2^
Cebadores RT-LAMP	F3	2 μ M	0,2 μ M	5'-AACACAAGCTTTCGGCAG-3'	5'-TGGCTACTACCGAAGAGCT-3·
Gen N SARS-CoV-2	B3	2 μ M	0,2 μ M	5'-GAAATTTGGATCTTTGTCATCC-3'	5'-TGCAGCATTGTTAGCAGGAT-3'
	BL	8 μ M	0,8 μ M	5'-TTCCTTGTCTGATTAGTTC-3'	5'-GGACTGAGATCTTTCATTTTACCGT-3'
	FL	8 μ M	0,8 μ M	5'-ACCTTCGGGAACGTGGTT-3'	5'-ACTGAGGGAGCCTTGAATACA-3'
	FIP	16 μ M	1,6 μ M	5'-TGCGGCCAATGTTTGTAATCAGC	5'-TCTGGCCCAGTTCCTAGGTAGTCCAGAC
				CAAGGAAATTTTGGGGAC-3'	GAATTCGTGGTGG-3'
	FIB	16 μ M	1,6 μ M	5'-CGCATTGGCATGGAAGTCACTTT	5--AGACGGCATCATATGGGTTGCACGGGTG
				GATGGCACCTGTGTAG-3'	CCAATGTGATCT-3'
Cebadores RT-qPCR	Cebador delantero-gen E			5'ACAGGTACGTTAATAGTTAATAGCGT3	
Gen E	Cebador inverso-gen E			5 ´ACAGGTACGTTAATAGTTAATAGCGT3	
	sonda TaqMan CAL FLUOR RED-gen E			610-5'ACACTAGCCATCCTTACTGCGCTTCG3'-BBQ-2	
	cebador delantero			5 ´-AGATTTGGACCTGCGAGCG-3 ´	
	cebador inverso			5 ´-GAGCGGCTGTCTCCACAAGT-3 ´	
	sonda TaqMan			HEX-5 ´TTCTGACCT-Nova-GAAGGCTCTGCGCG3 ´-BHQ-1	

La RT-qPCR se practicó con el equipo CFX96™ Bio-Rad, bajo las siguientes condiciones de amplificación: 50 °C por 20 minutos, seguido de 95 °C por 3 minutos, y finalizando con 45 ciclos de 95 °C por 15 s y 58 °C por 30 s.

### RT-LAMP

**Figura 3:**
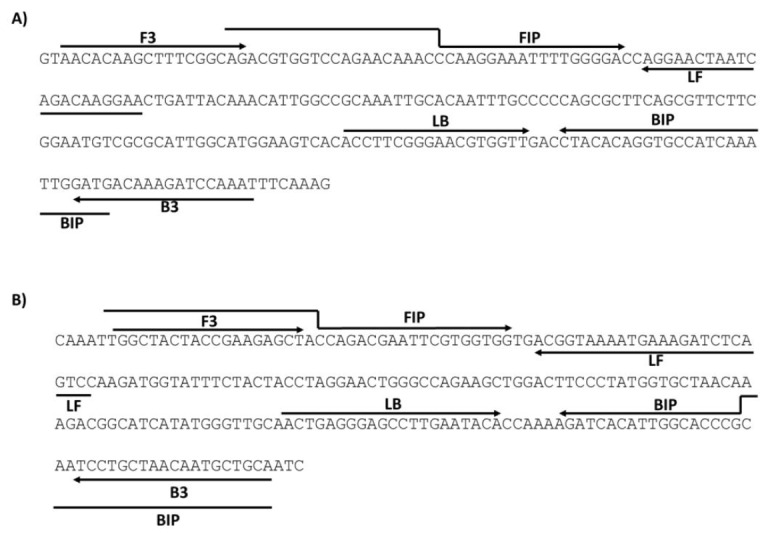
Localización de los sets de cebadores en el gen N del SARS-CoV-2. F3: cebador sentido, B3: cebador inverso o antisentido; FIP: cebador sentido interno; BIP: cebador inverso interno; LF: cebador lazo sentido; LB: cebador lazo inverso

Esta técnica de amplificación isotérmica se preparó con el kit Warmstart colorimetric LAMP 2X Master Mix (DNA & RNA) (NEB), siguiendo las recomendaciones de la casa comercial. En ella, se emplea un conjunto de seis cebadores para lograr la amplificación mediada por lazos. En este estudio de validación clínica, se emplearon los cebadores reportados por Broughton, *et al*. ^18^, y por Lalli, *et al*. ^19^; ambos juegos de cebadores amplifican un segmento del gen *N* del SARS-CoV-2, con 229 pares de bases para el primero y con 217 pares para el segundo. Como control positivo, en las reacciones de RT-LAMP se incluyó un plásmido que tiene clonado un fragmento del gen *N* del SARS-CoV-2, adquirido de la casa comercial New England Biolabs (NEB, Germany). Los cebadores usados en este estudio fueron sintetizados en Macrogen (Korea) (cuadro 1) (figura 3).

Antes de cada reacción LAMP, se preparó una mezcla 10X de cebadores de único uso y se procedió a realizar una corta incubación a 95 °C por 5 minutos, para eliminar cualquier formación de estructuras secundarias que pudieran afectar el cambio de color de la reacción. La concentración final de los cebadores en la reacción, después de agregar los demás reactivos, fue de 1X (cuadro 1).

La reacción LAMP se preparó empleando 10 μΙ del reactivo colorimétrico Warmstart colorimetric LAMP 2X Master Mix (DNA & RNA), 5 μl de agua, 3 μΙ del ARN previamente extraído y 2 μl de la mezcla maestra 10X del respectivo set de cebadores que anteriormente había sido incubado a 95 °C. La mezcla de cebadores 10X se adicionaba siempre en la tapa del tubo de la reacción, con la finalidad de evitar su degradación, así como amplificaciones inespecíficas que pudiesen empezar antes de la temperatura óptima de amplificación.

La reacción se iniciaba con un *spin* de los tubos a 65 °C por 30 minutos, en un termociclador (SimpliAmp Thermal Cycler™, Thermo Fisher Scientific). Transcurrido el tiempo de incubación, se visualizaba el resultado por colorimetría y se registraba fotográficamente con la cámara de un teléfono celular. El cambio de color de rosa a amarillo era indicativo de una reacción positiva, mientras que, si el color rosa se mantenía en la reacción, era señal de un resultado negativo para SARS-CoV-2 (figuras 1B y 2).

Para practicar la RT-LAMP en 36 muestras sin previa extracción de ARN, el protocolo fue bastante similar, con la siguiente modificación: la muestra provenía directamente del medio de transporte viral con un previo paso de incubación a 95 °C por 5 minutos. De ese volumen inactivado por calor, se utilizaron 3 μl para la reacción de RT-LAMP en lugar de ARN extraído con estuche comercial.

### Análisis

Para el análisis estadístico de los resultados, se empleó el programa estadístico Rstudio (versión 1.2.50033). La especificidad de la prueba RT- LAMP se calculó de la fracción de muestras con RT-qPCR negativas que también resultaron negativas en las pruebas de RT-LAMP. La sensibilidad Se expresó como la fracción de muestras que resultaron positivas en la qRT- PCR y, también, en la RT-LAMP. Se calcularon el valor predictivo positivo y el negativo, además del índice kappa (κ) para determinar la concordancia de los resultados de la RT-LAMP con los de la RT-qPCR.

## Resultados

En este trabajo se analizaron 153 muestras de individuos con sospecha de COVID-19, que asistieron a la Unidad de Genética y Biología Molecular de la Universidad Simón Bolívar entre los meses de junio de 2021 y enero de 2022. En 117 muestras se extrajo el ARN a partir de hisopados nasofaríngeos. De estas, 60 resultaron negativas, y 57 resultaron positivas para SARS-CoV-2 según la RT-qPCR (cuadro 2).

Las amplificaciones cuyo umbral de ciclos (*Cycle Threshold*, Ct) estuviera en un rango de 12 a 38, se consideraron positivas para SARS-CoV-2 ^(20)^. En estas mismas muestras, la RT-LAMP fue positiva en 58 y negativa en 59 (cuadro 2). Finalmente, el rendimiento de la RT-LAMP se evaluó bajo condiciones menos complejas en 36 muestras de hisopado nasofaríngeo, es decir, sin extracción previa de ARN (cuadro 2).

**Cuadro 2:** Comparación del rendimiento de los dos sets de cebadores y muestras sin previa extracción de ARN, para la ejecución de la prueba RT-LAMP y la RT-qPCR

Comparación de los resultados RT-qPCR con RT-LAMP		Positivo	Negativo	Total
Set de Broughton	RT-qPCR	35	37	72 117
	RT-LAMP	41	31	
Set de Lalli	RT-qPCR	22	23	45
	RT-LAMP	17	28	
Sin extracción de ARN	RT-qPCR	19	17	46
	RT-LAMP	20	16	

Para validar la prueba RT-LAMP en muestras de hisopado nasofaríngeo, se utilizaron los dos sets ya mencionados, cada uno con seis cebadores y ambos diseñados para amplificar un fragmento del gen *N* del SARS-CoV-2, que en adelante se denominan el “set de Broughton” y el “set de Lalli”. Los cebadores del primero reconocen una porción C-terminal del gen *N*^18^ y los del segundo set fueron diseñados contra la región N-terminal del gen *N*^19^ (figura 3 y cuadro 1).

Todas las reacciones fueron ejecutadas a una única temperatura de 65 °C por un tiempo de 30 minutos. En seguida, se registraron fotográficamente con la cámara de un teléfono celular (figuras 1 y 4).

**Figura 4:**
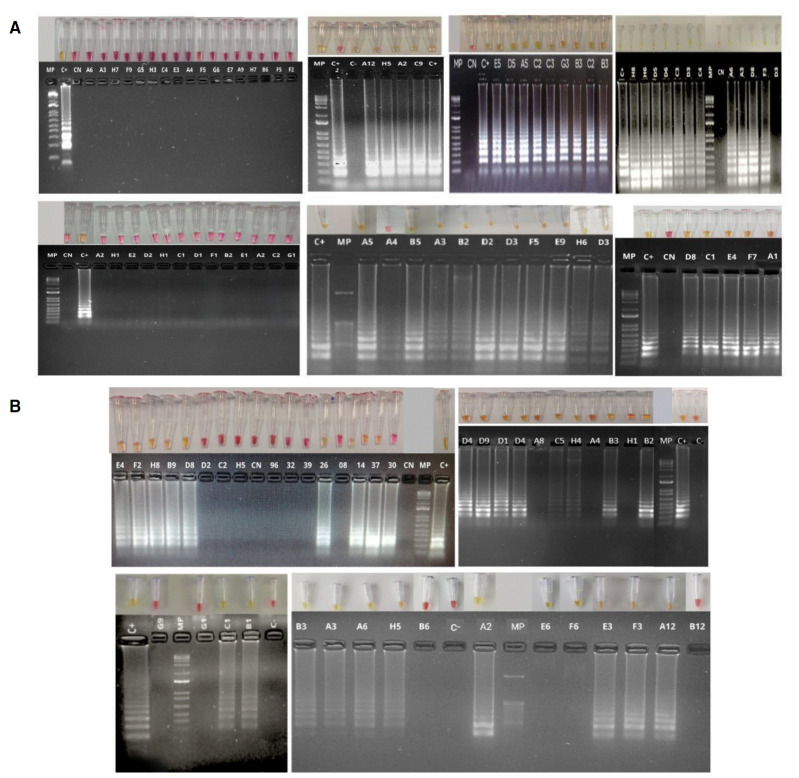
Detección colorimétrica de SARS-CoV-2 por RT-LAMP; empleando dos sets de cebadores, Broughton (A) y Lalli (B). Se recolectaron 117 muestras de ARN purificado a partir de hisopado nasofaríngeo de individuos sintomáticos para COVID-19. La reacción fue ejecutada a una temperatura de 65 °C durante 30 minutos, usando el estuche comercial Warmstart colorimetric LAMP 2X Master Mix (DNA & RNA) (NEB) en 20 μΙ de volumen final. La reacción de RT-LAMP tenía como diana el gen *N* del SARS-CoV-2. Las muestras negativas para SARS-CoV-2 se muestran en color rosa y las muestras positivas se visualizan por un cambio de color de rosa a amarillo. Los amplicones producto de la reacción de amplificación fueron separados en un gel de agarosa al 2 % y teñidos con SYBR Safe (Invitrogen). El patrón de tipo escalera observado en las muestras positivas, es coherente con una amplificación positiva y el subsecuente viraje de color en las muestras con presencia del SARS-CoV-2, en tanto que los productos de amplificación están ausentes en las muestras negativas. CP: fragmento sintético del gen *N* del SASR-CoV-2. CN: reacciones incubadas con agua de grado para biología molecular en lugar de ARN.

Una característica de la prueba LAMP, que se sustenta en su propio fundamento, es la generación de productos de diferentes masas moleculares que pueden visualizarse en un gel de agarosa como una escalera, patrón típico de bandeo de la LAMP (figura 1C) ^21^. Las RT-LAMP positivas muestran un patrón de bandeo de diferentes masas moleculares, correspondiente a los diferentes fragmentos obtenidos por amplificación isotérmica mediada por lazos (LAMP) (figura 4). Al contrario, en las muestras negativas no se evidencia ningún patrón de amplificación.

De las 117 muestras de ARN extraídas a partir de hisopados nasofaríngeos, los resultados de la RT-LAMP en 17 no correspondieron con los obtenidos por RT-qPCR, pero en las otras 100 muestras, sí lo hicieron (cuadro 2).

Para determinar su rendimiento de manera individual, se calcularon la sensibilidad y la especificidad de ambos sets de cebadores dirigidos contra el gen *N*. Para la reacción RT-LAMP con el set de Broughton, se incluyeron 72 muestras de ARN, de las cuales 64 fueron concordantes entre RT-LAMP y qRT-PCR, y 8 fueron discordantes. Hubo un falso negativo y 7 falsos positivos, de acuerdo con lo observado en la tabla de contingencia. Para el set de Lalli, se analizaron 45 muestras de ARN: 22 muestras positivas y 23 muestras negativas para RT-qPCR, en seis de las cuales no correspondieron con los resultados de la RT-LAMP (cuadro 3).

**Cuadro 3:** Tabla de contingencia RT-LAMP Vs. RT-qPCR correspondiente a los dos sets de cebadores dirigidos contra el gen *N* del SARS-CoV-2, set de Broughton y set de Lalli, y muestras sin extracción de ARN previa

Tabla de contingencia				
Set de Broughton	RT-LAMP	RT-qPCR		Total
		Positivos	Negativos	
	Positivos	34	7	41
	Negativos	1	30	31
	Total	35	37	72
Set de Lalli	RT-LAMP	RT-qPCR		Total
		Positivos	Negativos	
	Positivos	22	5	27
	Negativos	1	17	18
	Total	23	22	45
Sin extracción de ARN	RT-LAMP	RT-qPCR		Total
	Positivos	18	2	20
	Negativos	1	15	16
	Total	19	17	36

Con base en lo anterior, se encontró que la reacción colorimétrica RT- LAMP, con los cebadores del set de Broughton para detección del SARS- CoV-2, tuvo una sensibilidad de 0,97 y una especificidad del 0,81. El valor predictivo positivo fue de 0,83 y el valor predictivo negativo fue de 0,97. Por otro lado, empleando el set de Lalli para la RT-LAMP, la sensibilidad e de 0,96 y la especificidad fue de 0,77, con un valor predictivo positivo de 0,81 y valor predictivo negativo de 0,94 (cuadro 4).

**Cuadro 4:** Validación de RT-LAMP para diagnóstico del gen N de SARS-CoV-2. Sensibilidad, especificidad; valores predictivos de muestras positivas y negativas; concordancia por conformidad mediante el índice kappa (κ) con intervalo de confianza del 95 %.

Set de cebadores	Sensibilidad	Especificidad	Valor predictivo positivo	Valor predictivo negativo	Índice kappa	Po	Pe
Set de Broughton	0,97 (0,85-1,00)	0,81 (0,65-0,92)	0,83 (0,68-0,93)	0,97 (0,83-1,00)	0,778	0,88	0,49
Set de Lalli	0,96 (0,78-1,00)	0,77 (0,55-0,92)	0,81 (0,62-0,94)	0,94 (0,73-1,00)	0,73	0,86	0,50
Sin extracción de ARN	0,95 (0,74-1,00)	0,88 (0,64-0,99)	0,90 (0,68-0,99)	0,94 (0,70;-1,00)	0,83	0,91	0,50

Índice κ: valores de 0>0,2 mínima concordancia, 0,2>0,4 escasa concordancia, 0,4>0,6 moderada, 0,6>0,8 buena concordancia, 0,8>1 muy buena concordancia

Además, para los dos sets de cebadores evaluados, se analizó la concordancia entre los resultados para la técnica RT-LAMP usando la índice kappa (κ) ^22^.

El índice κ, para la reacción de RT-LAMP empleando el set de Broughton, fue de 0,76, mientras que, para el set de Lalli fue de 0,73 (cuadro 4). Estos valores demuestran que ambos grupos de cebadores le permitieron a la técnica RT-LAMP discriminar entre muestras positivas y negativas para SARS-CoV-2, con una buena concordancia entre los dos métodos diagnósticos, RT- qPCR y RT-LAMP, evidenciando su potencial diagnóstico para el SARS-CoV-2.

El desarrollo actual de técnicas de diagnóstico de agentes patógenos apunta a la implementación de técnicas que permitan su utilización a gran escala y en laboratorios con infraestructura limitada, así como, favorecer su uso y utilidad en los puntos de atención primaria. En ese contexto, la prueba RT-LAMP ha demostrado buenos resultados al incluir ácidos nucleicos purificados, así como, muestras directas sin previa extracción de ARN ^19^^,^^23^.

**Figura 5:**
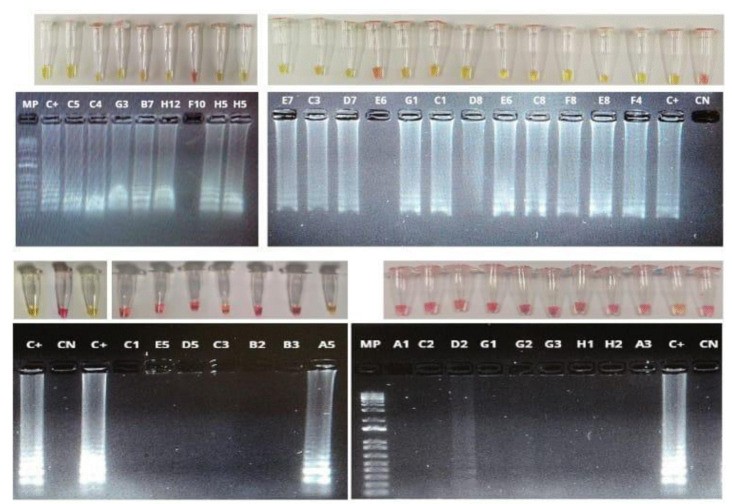
Detección colorimétrica de SARS-CoV-2 por RT-LAMP en muestras de hisopado nasofaríngeo, sin previa extracción de ARN. Se precalentaron 36 muestras de hisopado nasofaríngeo durante 5 minutos a 95 °C; posteriormente, se agregaron 3 μl de muestra a la reacción RT-LAMP usando el estuche comercial Warmstart colorimetric LAMP 2X Master Mix (DNA & RNA) (NEB) en 20 μl de volumen final. La RT-LAMP tenía como diana el gen N del SASR-CoV-2. Las muestras negativas para este virus se muestran en color rosa y las muestras positivas se visualizan por un cambio de color de rosa a amarillo. Los amplicones producto de la reacción de amplificación fueron resueltos en un gel de agarosa al 2 % y teñidos con SYBR Safe (Invitrogen). Se muestra el patrón típico de amplificación de la técnica RT- LAMP en muestras positivas y el cambio de color en la reacción; en muestras negativas, la mezcla reactiva permanece de color rosa y no se observan bandas en el gel electroforético. CP: fragmento sintético del gen N del SASR-CoV-2. CN: reacciones incubadas con agua de grado para biología molecular en lugar de muestras de hisopado.

El siguiente paso en la validación de la técnica de RT-LAMP fue evaluar su rendimiento empleando el set de Broughton en muestras sin previa extracción de ARN. Para ello, se validaron 36 muestras de hisopado nasofaríngeo de individuos sintomáticos con sospecha de COVID-19, a las cuales se les hizo un pretratamiento térmico por 5 minutos a 95 °C, sin ningún tipo de proteasa ni químicos adicionales. Posteriormente, se practicó la RT-LAMP, siguiendo las mismas condiciones descritas anteriormente, para la amplificación del gen *N* del SARS-CoV-2. Los resultados obtenidos arrojaron buenos niveles de sensibilidad y especificidad: 0,94 y 0,88, respectivamente, inclusive con una índice κ superior a los análisis, usando el ARN extraído de las muestras de hisopado nasofaríngeo (figura 5 y cuadro 4).

## Discusión

La pandemia de COVID-19 motivó al gobierno nacional a fortalecer las capacidades científicas y tecnológicas de los laboratorios de biología molecular del país, para apoyar el diagnóstico molecular del SARS-CoV-2 y otros agentes patógenos de interés en salud pública. No obstante, en Colombia el diagnóstico molecular de agentes infecciosos sigue siendo centralizado, debido a los pocos laboratorios que cuentan con infraestructura y equipos especializados para realizar dichas pruebas.

Los métodos de diagnóstico rápido y a gran escala son necesarios para prevenir y contrarrestar la propagación, no solo del SARS-CoV-2 en esta pandemia, sino también de brotes por otros agentes emergentes y reemergentes. En estos casos, pruebas como la LAMP se convierten en alternativas viables para apoyar el diagnóstico molecular de patógenos en lugares apartados con poco desarrollo, en donde no es posible confirmar el diagnóstico de ciertas enfermedades y las muestras de los pacientes deben enviarse a otros laboratorios, lo cual retrasa la obtención de los resultados y, por consiguiente, el tratamiento del paciente.

Debido a su facilidad de ejecución, la RT-LAMP está siendo ampliamente usada para detectar un gran número de agentes patógenos. Algunas de las ventajas inherentes a la técnica, incluyen: a) rapidez de amplificación de ácidos nucleicos, tanto de ADN como de ARN; b) buena especificidad debido al uso de seis cebadores; y c) gran tolerancia a los inhibidores ^24^. En el caso específico del SARS-CoV-2, se han reportado diversos protocolos en los que se utilizan varios sets de cebadores dirigidos contra diferentes genes del genoma viral, con diversa sensibilidad y especificidad ^18^^,^^19^^,^^25^^-^^27^. Características como velocidad, costo, tiempo de entrega del resultado, y la simplicidad de la ejecución de la prueba y de su lectura colorimétrica hacen de la técnica molecular RT-LAMP una solución efectiva para impulsar la capacidad de testeo, sobre todo en circunstancias como las actuales, en las cuales nuevas variantes virales con gran capacidad de transmisión están sobrepasando la capacidad de los laboratorios de referencia. Además, debido a que esta amplificación no requiere equipos sofisticados ni personal muy entrenado para su ejecución, es una excelente alternativa para implementarla en puntos estratégicos de atención en salud como aeropuertos, centros de salud locales, hospitales y otros.

La sensibilidad y especificidad de esta prueba en el diagnóstico del SARS- CoV-2 nos permite sugerirla como una alternativa viable para el diagnóstico rápido de la COVID-19.

Con relación al set de Broughton, los autores reportaron valores de sensibilidad y especificidad superiores a los reportados en el presente trabajo. Sin embargo, ellos emplearon una técnica de diagnóstico basado en el sistema CRISPR-Cas12a para detección del SARS-CoV-2. La RT-LAMP solo se usó para la amplificación inicial de la diana; la lectura o análisis de los resultados se hizo por fluorimetría, una metodología mucho más sensible en comparación con la simple inspección visual, lo que podría explicar dichos valores ^18^. El segundo set de cebadores validado en este estudio fue el reportado por Lalli, *et al*., quienes, además, incluyen un protocolo para detectar el genoma del SARS-CoV-2 directamente en muestras de saliva, sin necesidad de extraer antes ARN ^19^. Se ha demostrado que la saliva es un biofluido mucho mejor que los lavados nasofaríngeos para diagnosticar este virus, probablemente por su estabilidad y la mayor carga viral ^28^.

Una potencial limitación de este trabajo es el uso de ARN como matriz de diagnóstico, lo que no siempre es posible en centros de atención con infraestructura limitada. No obstante, según otros reportes previos, es posible adaptar la extracción de ARN mediante protocolos basados en perlas magnéticas, sin necesidad de centrífugas u otros equipos complejos, lo cual permite practicar a gran escala esta técnica diagnóstica ^29^.

En el presente estudio, se usaron muestras sin previa extracción de ARN viral, las cuales se sometieron a altas temperaturas por un corto período, para evaluar el rendimiento de uno de los sets de cebadores, escogido de forma aleatoria (set de Broughton). El protocolo de la RT-LAMP utilizado directamente en muestras sin previa extracción de ARN, demostraron gran sensibilidad y especificidad, 0,947 y 0,88, respectivamente (figura 5) (cuadro 4).

**Figura 6:**
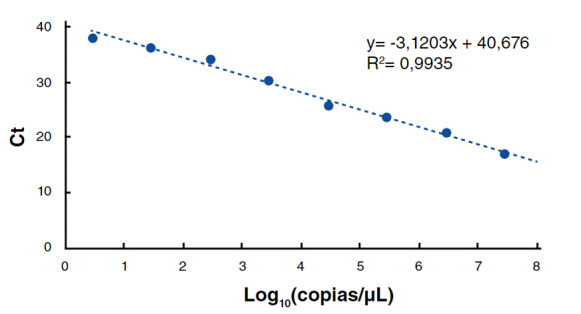
Curva de calibración número de copias gen E del SARS-CoV-2. Se confirmó el número de copias encontrado a partir de la fórmula: Número de copias (moléculas) = (Xng * 6.0221 * 10^23^ moléculas/mol) ^ ((N *660 g/mol) * 1 * 10 ^9^ng/g) Donde, X = concentración del amplicón (ng); N = longitud del amplicón dsDNA 660 g/mol = masa media de 1 pb de dsDNA

Se evaluó la sensibilidad de la técnica RT-LAMP para la detección del SARS-CoV-2 en relación con el número de copias virales en la muestra. Para ello, los valores CT (umbral de ciclo de amplificación) obtenidos en RT-qPCR se convirtieron a números de copias a partir de una curva de calibración de PCR con el plásmido pUC RdRp-E 3180pb (este plásmido fue donado gentilmente por el Dr. Jaime Castellanos de la Universidad El Bosque, y contiene un inserto con los genes virales E y RdRP de SARS-CoV2). Se realizaron diluciones seriadas en base 10 del plásmido a partir de una concentración inicial de 10 ng/μl, obteniendo por PCR el valor CT. El número de copias inicial del plásmido se halló en función de su longitud en pares de bases (pb), concentración inicial y %G/C (50%) según la fórmula de la Figura 6. De esta manera, se relacionó CT (basado en concentración) con el número de copias. El número de copias se determinó por medio de la ecuación lineal, Y = (-3.120X) + 40.676, donde X es el número de copias y Y el umbral de ciclos de amplificación, para un R^2^ = 0,993. El número de copias estará expresado en log_10_ por volumen de muestra (figura 6).

Respecto a la sensibilidad de la prueba RT-LAMP en relación con la carga viral, se encontró que, para ambos sets de cebadores utilizados, la sensibilidad de la técnica llega a 1 con un umbral de ciclos entre 25 y 35, es decir, en muestras cuya carga viral sea superior a 13 copias/μΙ y alcance las 21.130 copias/μl. Sin embargo, la sensibilidad cae significativamente, a 0,75 en el set Broughton y a 0,50 en el de Lalli, cuando la carga viral es muy inferior a 13 copias/μL o dicho umbral es mayor de 35.

Teniendo en cuenta la dinámica de la pandemia y la aparición de variantes con un mayor potencial de diseminación, consideramos que la prueba RT- LAMP reportada en este trabajo se convierte en una excelente alternativa para el diagnóstico del SARS-CoV-2 en Colombia, con la ventaja de poder utilizarse en territorios con escasez de recursos (sin necesidad de equipos sofisticados ni personal entrenado), así como en lugares con grandes tasas de transmisibilidad, como los aeropuertos.

Respecto a los costos, la prueba RT-LAMP es significativamente más económica que la RT-qPCR. Por muestra, la RT-LAMP tiene un costo aproximado de USD$ 7,95 si se utiliza el ARN, valor que cae a menos de USD$ 3,85 si se practica directamente sin extracción previa de ARN viral a partir de un hisopado nasofaríngeo. Por el contrario, la reacción de PCR en tiempo real tiene un valor en el mercado que oscila entre USD$ 41 y USD$ 56, un costo considerablemente más elevado que el estimado para la RT-LAMP.

Al momento del inicio de la pandemia por Covid-19, muy pocos laboratorios a nivel nacional y local cumplían con las condiciones de infraestructura y equipamiento para detectar este agente patógeno y, claramente, fueron insuficientes en el momento crítico de la pandemia cuando los casos superaban los miles por día. En la actualidad, en el departamento del Atlántico (Colombia), además de los laboratorios clínicos particulares, existen tres laboratorios públicos avalados por el Instituto Nacional de Salud para apoyar el diagnóstico del SARS-CoV-2.

El desarrollo de métodos diagnósticos de principio isotérmico sería una alternativa donde la infraestructura y la capacidad de adecuación sean limitadas. Además, pueden servir como prueba de tamizaje molecular, con la ventaja de poder llevarse a sitios con gran flujo de personas e, incluso, adaptarse rápidamente para identificar otros patógenos.

En conclusión, se reporta una metodología isotérmica para la amplificación del genoma del SARS-CoV-2 en muestras de hisopado nasofaríngeo con gran sensibilidad y aceptable especificidad. Esta prueba puede adaptarse y practicarse de forma rápida en instituciones con escasos recursos, para apoyar masivamente en la pandemia actual por COVID-19.

## References

[1] Dong E, Du H, Gardner L. (2020). An interactive web-based dashboard to track COVID-19 in real time. Lancet Infect Dis.

[2] Chiem K, Morales-Vásquez D, Park JG, Platt RN, Anderson T, Walter MR (2021). Generation and characterization of recombinant SARS-CoV-2 expressing reporter genes. J Virol.

[3] Jegerlehner S, Suter-Riniker F, Jent P, Bittel P, Nagler M. (2021). Diagnostic accuracy of a SARS- CoV-2 rapid antigen test in real-life clinical settings. Int J Infect Dis.

[4] Maricic T, Nickel O, Aximu-Petri A, Essel E, Gansauge M, Kanis P (2020). A direct RT- qPCR approach to test large numbers of individuals for SARS-CoV-2. PLoS ONE.

[5] Mustafa MI, Makhawi AM. (2021). SHERLOCK and DETECTR: CRISPR-Cas systems as potential rapid diagnostic tools for emerging infectious diseases. J Clin Microbiol.

[6] Xiong D, Dai W, Gong J, Li G, Liu N, Wu W (2020). Rapid detection of SARS-CoV-2 with CRISPR-Cas12a. PLOS Biol.

[7] Ghosh P, Chowdhury R, Hossain ME, Hossain F, Miah M, MdU Rashid Evaluation of recombinase-based isothermal amplification assays for point-of-need detection of SARS- CoV-2 in resource-limited settings. Int J Infect Dis.

[8] Liang Y, Lin H, Zou L, Zhao J, Li B, Wang H (2021). CRISPR-Cas12a-based detection for the major SARS-CoV-2 variants of concern. Microbiol Spectr.

[9] Ma L, Yin L, Li X, Chen S, Peng L, Liu G (2022). A smartphone-based visual biosensor for CRISPR-Cas powered SARS-CoV-2 diagnostics. Biosens Bioelectron.

[10] Nagamine K, Hase T, Notomi T. (2002). Accelerated reaction by loop-mediated isothermal amplification using loop primers. Mol Cell Probes.

[11] Tanner NA, Zhang Y, Evans TC (2015). Visual detection of isothermal nucleic acid amplification using pH-sensitive dyes. BioTechniques.

[12] Lamb LE, Bartolone SN, Ward E, Chancellor MB (2020). Rapid detection of novel coronavirus/ Severe Acute Respiratory Syndrome Coronavirus 2 (SARS-CoV-2) by reverse transcriptionloop-mediated isothermal amplification. PLoS ONE.

[13] Fischbach J, Xander NC, Frohme M, Glõkler JF. (2015). Shining a light on LAMP assays. A comparison of LAMP visualization methods including the novel use of berberine. BioTechniques.

[14] Park GS, Ku K, Baek SH, Kim SJ, Kim SI, Kim BT (2020). Development of reverse transcription loop-mediated isothermal amplification assays targeting severe acute respiratory syndrome coronavirus 2 (SARS-CoV-2). J Mol Diagn.

[15] Notomi T. (2000). Loop-mediated isothermal amplification of DNA. Nucleic Acids Res.

[16] Aliotta JM, Pelletier JJ, Ware JL, Moran LS, Benner JS, Kong H. (1996). Thermostable Bst DNA polymerase I lacks a 3’-->5’ proofreading exonuclease activity. Genet Anal Biomol Eng.

[17] Corman VM, Landt O, Kaiser M, Molenkamp R, Meijer A, Chu DK (2020). Detection of 2019 novel coronavirus (2019-nCoV) by real-time RT-PCR.. Eurosurveillance.

[18] Broughton JP Deng X, YuG Fasching CL, Servellita V Singh J (2020). CRISPR-Cas12- based detection of SARS-CoV-2. Nat Biotechnol.

[19] Lalli MA, Langmade JS, Chen X, Fronick CC, Sawyer CS, Burcea LC (2021). Rapid and extraction-free detection of SARS-CoV-2 from saliva by colorimetric reverse-transcription loop-mediated isothermal amplification. Clin Chem.

[20] Aranha C, Patel V, Bhor V, Gogoi D. (2021). Cycle threshold values in RT-PCR to determine dynamics of SARS-CoV-2 viral load: An approach to reduce the isolation period for COVID-19 patients. J Med Virol.

[21] Soni S, Salhotra A, Suar M. (2015). Handbook of research on diverse applications of nanotechnology in biomedicine, chemistry, and engineering.

[22] Landis JR, Koch GG. (1977). The measurement of observer agreement for categorical data. Biometrics.

[23] Wei S, Kohl E, Djandji A, Morgan S, Whittier S, Mansukhani M (2021). Direct diagnostic testing of SARS-CoV-2 without the need for prior RNA extraction. Sci Rep.

[24] Francois P, Tangomo M, Hibbs J, Bonetti EJ, Boehme CC, Notomi T (2011). Robustness of a loop-mediated isothermal amplification reaction for diagnostic applications. FEMS Immunol Med Microbiol.

[25] Zhang Y, Odiwuor N, Xiong J, Sun L, Nyaruaba RO, Wei H (2020). Rapid molecular detection of SARS-CoV-2 (COVID-19) virus RNA using colorimetric LAMP. medRciv.

[26] Dao Thi VL, Herbst K, Boerner K, Meurer M, Kremer LP, Kirrmaier D (2020). A colorimetric RT- LAMP assay and LAMP-sequencing for detecting SARS-CoV-2 RNA in clinical samples. Sci Transl Med.

[27] Flynn MJ, Snitser O, Flynn J, Green S, Yelin I, Szwarcwort-Cohen M (2020). A simple direct RT-LAMP SARS-CoV-2 saliva diagnostic. medRciv.

[28] Savela ES, Viloria Winnett A, Romano AE, Porter MK, Shelby N, Akana R (2022). Quantitative SARS-CoV-2 viral-load curves in paired saliva samples and nasal swabs inform appropriate respiratory sampling site and analytical test sensitivity required for earliest viral detection. J Clin Microbiol.

[29] Klein S, Müller TG, Khalid D, Sonntag-Buck V, Heuser AM, Glass B (2020). SARS-CoV-2 RNA extraction using magnetic beads for rapid large-scale testing by RT-qPCR and RT-LAMP. Viruses.

